# Enhancing Photoprotection and Mitigating Ex Vivo Stratum Corneum Oxidative Stress: A Multifunctional Strategy Combining Rosmarinic Acid with UVB Filters

**DOI:** 10.3390/antiox14030274

**Published:** 2025-02-26

**Authors:** Pedro Ivo de Souza Macedo, Claudinéia Aparecida Sales de Oliveira Pinto, Camila Faustino Hiraishi, Gabriela de Argollo Marques, Cassiano Carlos Escudeiro, Fabiana Vieira Lima Solino Pessoa, João Gregório, Catarina Rosado, Maria Valéria Robles Velasco, André Rolim Baby

**Affiliations:** 1 Department of Pharmacy, Faculty of Pharmaceutical Sciences, University of São Paulo, São Paulo 05508-000, Brazil; 2 IPclin, Integrated Research, Jundiaí 13201-013, Brazil; 3 Department of Health Sciences, Faculty of Pharmacy, Federal University of Espírito Santo, São Mateus 29932-540, Brazil; 4 CBIOS, Universidade Lusófona’s Research Center for Biosciences & Health Technologies, 1749-024 Lisbon, Portugal

**Keywords:** ethylhexyl methoxycinnamate, octocrylene, ethylhexyl salicylate, sun protection factor, tape stripping

## Abstract

Exposure to ultraviolet (UV) radiation is a major contributor to skin injury, including sunburn, photoaging, and augmented risk of skin cancer, primarily through the generation of reactive oxygen species (ROS) that induce oxidative stress. Rosmarinic acid (RA), a natural phenolic compound with antioxidant and several other biological properties, has shown promise in mitigating such damage when incorporated into sunscreens. We evaluated RA’s possible interactions and potential to enhance the efficacy of three worldwide known UVB filters—ethylhexyl methoxycinnamate (EHMC), octocrylene (OCT), and ethylhexyl salicylate (EHS). The performance of sunscreens with and without RA (0.1% *w*/*w*) was analyzed through in vitro and in vivo photoprotective assessments. The HPLC-TBARS-EVSC (high-performance liquid chromatography—thiobarbituric acid reactive substances—ex vivo stratum corneum) protocol, which quantified oxidative stress reduction in the human stratum corneum, was also used. The in vitro photoprotective assays showed that RA had distinct levels of interactions with the UVB filters. When associated with EHMC, RA exclusively acted in the UVB range (SPF-enhancing effect). Remarkably, for EHS, RA contributed to a higher efficacy profile in the total UV spectrum. OCT-RA was the sample that reached the highest critical wavelength value parallelly to OCT, boosting the in vivo SPF by more than 157% in comparison to OCT. However, its in vitro SPF performance was not affected by the RA addition, being comparable to OCT, EHS, and EHS-RA. Furthermore, the HPLC-TBARS-EVSC protocol highlighted RA’s ability to reduce lipid peroxidation, with OCT-RA exhibiting the most notable protective effect. These findings underscore RA’s potential as a multifunctional additive in sunscreen systems, enhancing both photoprotection and oxidative stress mitigation.

## 1. Introduction

Awareness of ultraviolet (UV) radiation-induced skin damage—ranging from sunburn and photoaging to an increased risk of skin cancer—along with an understanding of the underlying mechanisms driving these injuries, has spurred interest in developing multifunctional sunscreens. These formulations aim to provide protection against both UVA and UVB rays, while also offering antioxidant properties. Such features are critical, as skin exposure to UV radiation stimulates the generation of reactive oxygen species (ROS), creating an oxidative imbalance. This leads to lipid peroxidation, compromising cell membranes, proteins, DNA, and other detrimental effects [1,2].

UV filters are the key active ingredients in sunscreens, shielding the skin from the deleterious action of UV radiation [3,4,5,6]. Our research group has been investigating multifunctional bioactive sunscreens and has observed that, while these molecules are undeniably critical for maintaining skin health, they often lack sufficient antioxidant activity to fully mitigate the oxidative stress experienced by the stratum corneum during UV exposure [7]. The aforementioned scenario highlights a gap in the UV filter market worldwide, creating a demand for antioxidant-active compounds that positively interact with sunscreen systems, and for robust protocols for establishing additional functional attributes of these molecules or systems.

Rosmarinic acid (RA) can enhance sunscreen systems by improving their safety, efficacy, or photostability [8,9]. RA is an antioxidant phenolic compound found in various plants, exhibiting a range of biological effects, including wound healing, anti-inflammatory, anti-angiogenic, antimutagenic, antibacterial and antiallergic activities, among others [10,11]. This broad spectrum of health benefits, particularly for skin care, makes RA a promising candidate for further investigation in combination with UV-absorbing molecules.

In this study, the possible synergies from the combination of RA with three isolated UVB filters—ethylhexyl methoxycinnamate, octocrylene, and ethylhexyl salicylate—were probed. The in vitro photoprotective efficacy of the sunscreen systems containing these UVB active molecules and RA was assessed using diffuse reflectance spectrophotometry with an integrating sphere. Additionally, the sun protection factor (SPF) of the samples was determined in vivo.

This is the first study to also apply the ex vivo non-invasive HPLC-TBARS-EVSC (high-performance liquid chromatography—thiobarbituric acid reactive substances—ex vivo stratum corneum) protocol to evaluate the efficacy of sunscreen compounds. This protocol was used to study the protective effects of RA, alone and in combination with UVB filters, against oxidative stress in the tape-stripped stratum corneum of participants [12,13,14].

## 2. Materials and Methods

### 2.1. Sunscreen Samples

The UVB filters alone (ethylhexyl methoxycinnamate, EHMC; octocrylene, OCT; and ethylhexyl salicylate, EHS) or in association with the rosmarinic acid at 0.1% (*w*/*w*) were vehiculated into an oil-in-water emulsion previously reported by de Oliveira Bispo and coworkers [8]. The sunscreen samples’ qualitative and quantitative compositions are described in Table 1.

### 2.2. In Vitro Photoprotective Efficacy

The photoprotective efficacy of the samples was assessed in vitro by measuring their sun protection factor (SPF) and critical wavelength (nm) using a Labsphere UV2000S Ultraviolet Transmittance Analyzer (Labsphere, North Sutton, NH, USA). Each sample (1.3 mg/cm^2^) was evenly distributed on polymethylmethacrylate plates (n = 3) (Helioplate HD 6, Weneos, Lacroix Saint Ouen, France) and allowed to dry under light-protected conditions. The details of the procedure followed the method described [9,15]. The UV-2000 software (version 1.1.1.0) was used to calculate the SPF and critical wavelength values. The samples were assayed in replicates of three (n = 3).

### 2.3. In Vivo and Ex Vivo Assays

The subjects (women and men aged 18–60 years) were thoroughly informed about the study objectives and procedures and provided written consent by signing an informed consent form. Confidentiality (anonymity) was strictly maintained, and the participants were assured of their right to withdraw from the study at any time. Ethical approval was obtained from the Ethics Committee of the Faculty of Pharmaceutical Sciences at the University of São Paulo (number 080392/2021), and the study adhered to the principles of the Helsinki Declaration.

#### 2.3.1. In Vivo SPF Assessment

The in vivo SPF determination followed guidelines and procedures described in the specialized literature [8,16,17]. A total of ten participants with skin phototypes II and III were included in the trial. Each sample (2.00 ± 0.05 mg/cm^2^) was applied to designated untanned areas on the participants’ backs, which were cleaned using dry cotton. After drying, the treated areas were exposed to UV radiation using a Multiport 601 UV Solar Simulator (Solar Light Company, Glenside, PA, USA). Visual assessments of the irradiated skin were performed 20 ± 4 h post-exposure under standardized lighting conditions, and the SPF values were calculated.

#### 2.3.2. Ex Vivo Anti-Lipoperoxidative Efficacy by HLPC-TBARS-EVSC

This protocol involved eight participants, including five females and three males with skin phototypes ranging from II to V. To minimize external factors, the participants were instructed to avoid applying topical products to their volar forearms for 24 h before the experiment [18,19].

Rectangular areas (5.0 × 2.0 cm) were randomly marked on the volar forearm of each participant, matching the dimensions of the adhesive tape used (Scotch Magic™ Tape, 3M, Sumaré, SP, Brazil). The skin was prepped by cleaning with dry cotton, and the stratum corneum was collected using the tape stripping method. Six consecutive adhesive tape strips were applied and removed from each site, with the first strip discarded. A consistent and uniform pressure was maintained throughout the procedure to ensure reproducibility.

One site was designated as the control (non-treated and non-irradiated), while those from the other site underwent artificial UV in a solar simulator chamber at 5506 KJ·m^−2^ for 2 h under controlled temperature conditions (~35 °C) and within a wavelength range of 290–400 nm. The UV radiation was generated using an Atlas Suntest CPS+ simulator (Atlas Material Testing Technology, Mount Prospect, IL, USA) equipped with a 1500 W xenon lamp and a filter permitting wavelengths above 290 nm. The five adhesive tapes containing stratum corneum samples from each site (one non-treated and the others treated with the formulations) were transferred to separate Falcon tubes to extract the biological material that went through a chemical reaction to obtain the MDA-TBA_2_ (malondialdehyde–thiobarbituric acid) adduct, as previously described by Marques and coworkers [19] and Ruscinc and coworkers [20]. The MDA-TBA_2_ adduct was quantified by HPLC. This biomarker is indicative of lipid peroxidation in the human stratum corneum.

The HPLC analysis was conducted using a Shimadzu Prominence system (Kyoto, Japan) equipped with an SPD-M20A diode array detector and a CTO-20A column oven set to 30 °C. A reversed-phase C18 column (Luna 5 µm C18(2), 250 × 4.6 mm, Phenomenex, Torrance, CA, USA) was used, preceded by a SecurityGuard pre-column cartridge (10 × 3.0 mm, Phenomenex, USA). An isocratic elution mode was employed, with a mobile phase consisting of 35% methanol and 65% phosphate buffer (50 mM, pH 7.0). The flow rate was maintained at 1.0 mL/min, and sample injections of 40 µL were performed. Detection was carried out using the diode array detector set to 532 nm [18,21].

### 2.4. Statistical Treatments

The results of in vitro SPF, critical wavelength (nm), and in vivo SPF were described as mean ± standard deviation and subjected to one-way analysis of variance (ANOVA) performed by Minitab software (version 21.1.0). In addition, the Tukey post-test was applied. To assess differences in the area under the curve (AUC) of the MDA-TBA_2_ adduct for the non-irradiated and the irradiated stratum corneum among categories, the t-test for independent samples or the Mann–Whitney test were used, following the use of the Shapiro–Wilk test for normal distribution. Repeated measures ANOVA was used to assess the differences in non-irradiated and irradiated AUC across multiple categorical independent variables. Linear regression was used to assess the association of independent variables with the ratio of AUC. Descriptive and inferential statistics were performed in jamovi 2.3.21 (jamoviproject, Sydney, Australia). A significance level of 5% (*p* < 0.05) was used for all the statistical treatments.

## 3. Results and Discussion

In the in vitro photoprotective test (Table 2), the SPF values for the samples ranged from 4 to 14 for the isolated UVB filters and from 5 to 17 for those containing the RA association. The most effective isolated UVB molecule based on this parameter (*p*-value < 0.05) was EHMC (SPF = 14 ± 1), followed by OCT (SPF = 6 ± 1) and EHS (SPF = 4 ± 0). EHMC, also known as octinoxate, is a strong absorber of UVB radiation derived from cinnamic acid, with a λ max of 308–311 nm and a high ε value of 23,300 mol^−1^cm^−1^. The structure of cinnamic acid derivatives, favoring the UVB absorbance property, features an unsaturated bond between the aromatic ring and the carboxyl group, thereby enhancing the efficacy of this class of UV filters [22,23]. Chemically, OCT is the 2-ethylhexyl ester of 2-cyano-3,3-diphenylacrylic acid, distinct from other cinnamates due to its ε value of 12,290 mol^−1^cm^−1^ and a peak around 303–304 nm. OCT demonstrates good photo and chemical stability, and a high critical wavelength compared to other UVB filters [22,24,25]. EHS (octisalate), a safe salicylic acid derivative commonly combined with other UV filters, exhibits low ε and weak effectiveness in absorbing UVB radiation, with a λ max of 306 nm [22,23,26]. Couteau and coworkers [26] studied the efficacy of several UV filters by determining the in vitro SPF values. Among the UVB-absorbing molecules selected herein, they found SPF values of 11.16 ± 0.41, 13.82 ± 1.08, and 2.7 ± 0.19 for EHMC, OCT, and EHS, respectively. While our EHMC SPF value (SPF = 14 ± 1) was comparable to their sample (SPF = 11.16 ± 0.41), making EHMC the most effective isolated UVB filter for us, their study positioned EHMC with an intermediate efficacy between OCT and EHS [26]. Peres and coworkers [25] also established the in vitro efficacy of EHMC and OCT, aligning with Couteau and coworkers [26]. However, they observed reduced SPF values, which could be attributed to differences in sample vehicles and the rate of sample mass application (mg/cm^2^) over the substrate surface. Our least active isolated UVB filter was EHS, which consistent with the results of Couteau and coworkers [26].

Upon incorporating RA at 0.1% *w*/*w* into the samples, we observed an enhancement of in vitro SPF values for EHMC-RA (SPF = 17 + 3) and EHS-RA (SPF = 7 + 1) (Table 2). However, OCT-RA exhibited a slight decay in SPF (5 + 0), albeit with a *p*-value > 0.05. Similar strategies, like Peres and coworkers [25], associated rutin, an antioxidant flavonoid, at 0.1% *w*/*w* to OCT, however, in a hydroalcoholic gel, and quantified the in vitro SPF by diffuse reflectance spectrophotometry with an integrated sphere. The authors found that the addition of rutin negatively interfered with OCT performance by diminishing the SPF, a result profile comparable with our trend for OCT-RA. Negative interferences on in vitro SPF due to natural compounds in sunscreen systems were reported in the literature, for example, rutin (0.1%) + avobenzone (AVO) (3.0%) + ethylhexyl dimethyl PABA (8.0%) [27]; *Passiflora incarnata* dry extract (1.68%) + EHMC (7.0%) + benzophenone-3 (2.0%) + TiO_2_ (2.0%) [28]; *Plantago lanceolata* hydroglycolic extract (2.78%) + EHMC (3.5%) + benzophenone-3 (1.0%) + TiO_2_ (1.0%) [28]; and *Vaccinium myrtillus* extract (5.0%) + methylene bis-benzotriazolyl tetramethylbutylphenol (6.0%) + OCT (1.5%) + TiO_2_ (2.5%) [7]. The quantitative and qualitative composition of the semisolid vehicle; the presence or absence of inorganic UV filters; the proportions of the organic UV filters, and the natural compound(s); and the photostability of the active ingredients could explain the distinct behaviors of the in vitro photoprotective efficacy among the systems [28].

Cândido and coworkers [9] employed concepts of quality by design (QbD) to prepare sunscreen systems containing EHMC and bemotrizinol (broad spectrum UV filter) associated with RA. The samples were planned through a design of experiments (DoE) to establish their in vitro antioxidant (DPPH test) and SPF (by diffuse reflectance spectrophotometry with integrated sphere) values along with functional photostability. The authors found that RA-containing formulations presented, in general, superior performance of in vitro antioxidant activity, and the highest one was for EHMC (7.5% *w*/*w*) + bemotrizinol (10.0% *w*/*w*) + RA (1.0% *w*/*w*). UV filters in the RA-free samples were not able to generate expressive antioxidant activity, with this finding corroborated by the scientific literature [25,27,29,30]. The presence of RA did not optimize the in vitro SPF of the EHMC and bemotrizinol; however, the authors observed that RA photostabilized the sunscreen system for the following association: EHMC (7.5% *w*/*w*) + bemotrizinol (10.0% *w*/*w*) + RA (1.0% *w*/*w*). Despite the fact that the authors have not noticed a positive nor negative impact on the RA association with the mixture of the UV filters in terms of in vitro SPF enhancement, the antioxidant compound offered the system a multi-benefit property by adding antioxidant action with functional photostability improvement.

The development process of sunscreen products must forecast the broadest possible UV absorption range that will be achieved, essentially, by the rationale selection of UVA, UVB, and broad spectrum UV filters. The critical wavelength (nm) is the parameter that will characterize the photoprotective formulation, concomitant with the in vivo SPF value, as a broad spectrum sunscreen [31,32,33]. In addition to the association of UV filters in general, natural/antioxidant compounds may contribute to this property by elevating its value on the final/finished product. The critical wavelengths for the isolated UV active compounds were 340, 348, and 326 nm, respectively, for EHMC, OCT, and EHS (Table 2). Our findings indicated that OCT had the best performance for the broad spectrum property, followed by EHMC and EHS. RA at 0.1% *w*/*w* improved the critical wavelength only of EHS-RA (326 to 331 nm), suggesting the addition of a protective effect against part of the UVA radiation for this UVB filter.

Peres and coworkers [25] established the critical wavelengths of hydroalcoholic gels containing EHMC or OCT added or not with rutin (0.1% *w*/*w*). They observed a promotion of this parameter value to the EHMC when associated with the flavonoid; however, for the OCT, rutin did not exert such an effect. The authors attributed the latter response to the inherent high critical wavelength value of the OCT (in comparison to other UVB filters), equivalent to our data, that possibly inhibited a positive interference of the flavonoid. Accordingly, RA neutrally interacted with OCT, which developed a similar result profile as rutin did, probably by a similar mechanism of action. Our results also indicated that RA interacted neutrally with the EHMC critical wavelength; however, there was an improvement in the UVB filter of the in vitro SPF.

Studies involving in vivo trials like our investigation have been reported. issues. For instance, Cândido and coworkers [34], in another research work, studied the RA potential in association with EHMC and bemotrizinol to mitigate cutaneous inflammatory response provoked by a topical application of methyl nicotinate solution in subjects. The release of prostaglandin D_2_ (PGE_2_)—which occurs also due to the incidence of UV radiation by the elevated presence of reactive oxygen species (ROS)—is part of the mechanism of action of nicotinic acid and its esters to cause local erythema by vasodilation of upper dermis peripheral capillaries. Taking into account the evidenced antioxidant action of RA [11,35], the authors expected to find a topical anti-inflammatory effect of the multifunctional sunscreen sample, although, according to their protocol, a decrease in the erythema formation nor its progression/intensity were observed. This behavior may be attributed to RA concentration. Psotova and coworkers [36] observed a decrease in protection activity against lipid peroxidation, among other parameters, of RA when in higher concentrations.

We set the RA concentration at 0.1% *w*/*w* since we previously evaluated its in vitro antioxidant activity by DPPH test, as an isolated compound, and we observed an efficacy of over 94% in scavenging the stable free radical, equivalent to the sample at 1.0% *w*/*w* (>95%) used by Cândido and coworkers [9,34]. We likewise selected only UVB filters of different efficacy profiles reported in the specialized literature [22,26] to allow the investigation of SPF in a safe experimental design and related efficacy parameters in the presence of RA. The scenario of the research on sunscreens/sunscreen systems/multifunctional sunscreens involves, predominantly, a mixture of UV-absorbing molecules [7,16,37,38,39] to achieve photoprotective action ranging from UVB to UVA radiation. We used this strategy to observe RA’s possible mechanism of action in a less complex matrix, while keeping the originality and innovation of this research in addition to the use of a novel protocol to establish ex vivo efficacy also linked to photoprotection, the HPLC-TBARS-EVSC.

**Figure 1:**
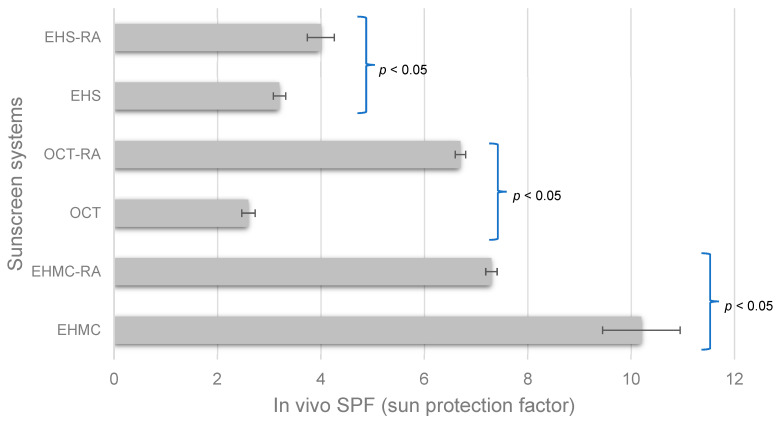
In vivo SPF (sun protection factor) the sunscreen systems (n = 10). EHMC—ethylhexyl methoxycinnamate; OCT—octocrylene; EHS—ethylhexyl salicylate; RA—rosmarinic acid.

Our in vivo SPF trial led us to the following main results for the isolated UVB filters: EHMC = 10.2 + 0.75; OCT = 2.6 + 0.13; and EHS = 3.2 + 0.12. Introducing RA to each UV-absorbing molecule yielded different values of the in vivo SPF, being 7.3 + 0.11, 6.7 + 0.10, and 4.0 + 0.26, respectively, for EHMC-RA, OCT-RA, and EHS-RA (*p*-value < 0.05) (Figure 1). The blank and the sample composed solely of RA at 0.1% *w*/*w* were not considered for this assay since they were not expected to protect the cutaneous tissue against potential harm during the artificial irradiation procedure.

The antioxidant ingredients improved the in vivo SPF for OCT-RA and EHS-RA but negatively impacted EHMC-RA, leading to decreased SPF (*p*-value < 0.05). Although this represents the first report, to the best of our knowledge, of a clinical assay involving RA with an isolated UVB filter, an unfavorable outcome for the photoprotection efficacy may be unexpected. SPF is an efficacy parameter recognized worldwide and its value, available on labels of products, means how protected the individual skin is before the formation of the perceptible erythema, i.e., sunburn. It is calculated as the quotient of the minimal erythema dose (MED) of the skin protected with sunscreen by the MED of the unprotected skin [40,41]. The erythema, a cutaneous acute response accompanied by edema, is the biological endpoint of the SPF test [17,42]; thus, considering a substance active enough to delay the perceptible erythema appearance, the SPF of the challenged sample would afford it a higher value, being correlated to a superior level of protection, if respecting the application conditions. RA is an antioxidant molecule that also acts as an anti-inflammatory agent by, for instance, reducing the synthesis of PGE_2_ (in a dose-dependent manner) and other inflammatory mediators [43,44].

De Oliveira Bispo and coworkers [8] developed emulsified systems composed of EHMC and AVO (a UVA filter) and determined their photoprotective efficacy. From the in vivo SPF trial, the addition of RA at 0.1% *w*/*w* to EHMC (10.0% *w*/*w*) + AVO (5.0% *w*/*w*) augmented the formulation efficacy by more than 40% compared to the control sunscreen (RA-free). Nonetheless, in another sample where the AVO concentration was reduced to 2.5% *w*/*w* (keeping EHMC at 10.0% *w*/*w*), in the presence of RA, notably, the formulation generated an SPF equivalent to the control, that had both UV filters at the maximum proportions (RA-free). The authors suggested, based on those findings allied to the determination of the critical wavelength [32,45,46,47,48] of the same samples in which no expressive improvement of this parameter was found, that RA could have contributed to SPF efficacy by a mechanism of action that involved not exclusively a UV absorption property. Besides the in vivo SPF-enhancing characteristic of RA when in the presence of EHMC (10.0% *w*/*w*) + AVO (5.0% *w*/*w*), this compound was not capable of significantly ameliorating the functional photostability of the sunscreen system, which contrasted with the findings of Marcílio Cândido and coworkers [9], who observed a photostabilization property of RA when associated with EHMC and bemotrizinol. The RA-containing samples lost between 34 and 42% of the in vitro SPF value calculated by the diffuse reflectance spectrophotometry with integrated sphere after 30 min of UV irradiation in a solar simulator. In comparison, the control had a subtle greater decay of 44% of this efficacy parameter.

The scenario of functional photostability tests applied to RA, up to now, allows us to suggest that its photostabilization property requires further investigation involving different ranges of concentrations, a variety of vehicles (lipophilic, hydrophilic, alcoholic, hydroalcoholic, emulsion, etc.), and more types of UV-absorbing compounds and their associations since this molecule showed, apparently, a case-by-case interaction profile for distinct UV filters/sunscreen systems. RA (0.1%) was not enough effective to maintain the stability performance of the known photounstable [23,49] mixture of EHMC and AVO [8]; however, it improved the photostability of the mixture of EHMC and bemotrizinol when the antioxidant compound was at 1.0% [29].

Obtaining sunscreens with safe and effective profiles, broad spectrum properties, and multifunctional characteristics is challenging, as well as the selection of tests of claim substantiations, which will require non-invasive (or minimally non-invasive) measurements of biomarkers, cutaneous attributes, and biological endpoints, preferentially, in participants. In addition to the previously described, there is the concern about developing accessible final products with pleasant sensorial properties, photo and chemical stability, and low environmental impact. To support the advances in the overall process of the development of sunscreens, innovative protocols must be investigated, allowing the reliable determination/quantification of more types and safer endpoints to establish safety and efficacy, reinforcing industry credibility and consumer care [19,42]. The HPLC-TBARS-EVSC protocol is an ex vivo non-invasive assay [18,19,50] in which the lipid peroxidation level of the tape-stripped stratum corneum, stressed or not by an artificial UV source, is quantified by HLPC as an MDA or MDA-TBA_2_ adduct [12,13,21], thus, affording data about the oxidative stress degree of the outermost layers of the epidermis. It is noteworthy to mention that this protocol did not expose the subjects to UV radiation.

Samples containing EHMC, OCT, or EHS, added or not with RA, demonstrated anti-lipoperoxidative efficacy against UV-induced oxidative stress of the tape-stripped stratum corneum quantified by our protocol (Table 3 and Table S1). The blank was ineffective in preventing the formation of lipid peroxides in the irradiated biological material. When the percentage difference analyzed these findings (i.e., comparing the baseline non-irradiated blank AUC versus the AUC from the treated and irradiated sites), we observed that RA-free formulations rose in the final AUC. In contrast, the ones added with RA presented values near zero, however, with *p*-values > 0.05. The OCT-RA sample promoted the best protection against the oxidative stress of the tape-stripped stratum corneum by meaningfully generating a reduction in the difference in percentage of the AUC (*p*-value = 0.036), i.e., OCT-RA demonstrated a better capacity to prevent lipoperoxidation of the outermost layers of the skin than EHMC-RA and EHS-RA.

Reactions derived from lipid peroxidation may be associated with the production of reactive species of nitrogen and/or oxygen, which are involved in many disorders [18]. Nevertheless, the formation of lipid peroxides can be prevented by the use of antioxidants, free radical scavengers, and singlet oxygen quenchers. Sánchez-Campillo and coworkers [51] determined the antioxidant activity of RA, as a purified rosmarinic acid extract from rosemary (*Rosmarinus officinalis* L.) leaves, by the auto-oxidation of linoleic acid through the MDA measurement using the TBARS test. The authors demonstrated, in function of time, that the purified RA extract delayed MDA formation to a greater extent than the *L*-ascorbic acid. According to our results, despite RA, the isolated UV filters also showed a protective effect against the lipid peroxidation of the tape-stripped stratum corneum; however, in the presence of the antioxidant compound (EHMC-RA, OCT-RA, and EHS-RA), a tendency of higher inhibition of the MDA-TBA_2_ adduct formation was observed (Table 3). The OCT-RA enhancement on the property to counteract the elevation of cutaneous oxidative stress could be related to the RA antioxidant and anti-inflammatory activities [37,51,52] in association with the UVB-absorbing efficacy of the OCT that resulted in an expressive delay of the erythema formation and the broad spectrum characteristic of this multifunctional sample, by its value of critical wavelength.

Our study presents strengths, particularly the incorporation of RA at 0.1% *w*/*w* into sunscreen prototypes, demonstrating multifunctional potential in enhancing photoprotection and mitigating oxidative stress in the human stratum corneum within a short exposure period (2 h of skin contact). The ex vivo HPLC-TBARS-EVSC protocol, despite the limited number of participants, supported the hypothesis that RA effectively reduced lipid peroxidation in the stratum corneum, with the most pronounced effect observed in the formulation containing OCT, reinforcing its role in preventing UV-induced oxidative damage. Despite these advantages, certain limitations should be acknowledged. The fixed RA concentration, while effective, may not represent the optimal condition for synergy with the UVB filters, highlighting the need for further dose–response studies. Expanding the study to include a broader range of UV filters, covering UVA, UVB, and broad spectrum protection, would provide a more comprehensive assessment of RA’s potential. Additionally, scalability remains a challenge, as ensuring RA associations’ stability and efficacy in large-scale production requires further investigation. Consumer acceptance is another critical factor, as RA incorporation may impact formulation costs, regulatory compliance, and market viability, all of which must be carefully evaluated for successful commercial implementation.

## 4. Conclusions

OCT-RA, among all efficacy parameters we have investigated, was the sample that reached the highest critical wavelength value parallelly to OCT (the isolated molecule) and the highest enhancement of the in vivo SPF (increase of more than 157% in comparison to OCT); however, its in vitro SPF performance was not affected by the RA addition, being comparable to OCT, EHS, and EHS-RA. Through the data of the HPLC-TBARS-EVSC protocol, the addition of RA to the different formulations, except for the blank sample, did not result in significant decreases in the AUC of the irradiated stratum corneum samples. However, OCT-RA developed a substantial reduction in this parameter, demonstrating the differentiated effectiveness of this sunscreen system in mitigating the stratum corneum lipid peroxidation compared with EHMC, EHMC-RA, EHS, and EHS-RA.

## Figures and Tables

**Table 1:** Qualitative and quantitative compositions (%, *w*/*w*) of sunscreen systems.

Ingredients/Function	Proportions % (*w*/*w*)							
	EHMC	EHMC-RA	OCT	OCT-RA	EHS	EHS-RA	Blank	Blank-RA
Cetearyl alcohol (and) dicetyl phosphate (and) ceteth-10 phosphate (Crodafos CES)/Self-emulsifying base	4.0							
Isopropyl myristate/Emollient	5.0							
Rosmarinic Acid/Active ingredient (antioxidant compound)	0.0	0.1	0.0	0.1	0.0	0.1	0.0	0.1
Glycerin/Humectant	5.0							
Phenoxyethanol, methylparaben, butylparaben, ethylparaben, propylparaben and isobutylparaben/Preservative system	0.75							
Ammonium acryloyldimethyltaurate/VP copolymer (Aristoflex AVC)/Emulsifier polymer	1.0							
Ethanol/Co-solvent	5.0							
Purified water (aqua)/Solvent (vehicle)	q.s.							
Ethylhexyl methoxycinnamate/UVB filter	10.0	10.0	0.0	0.0	0.0	0.0	0.0	0.0
Octocrylene/UVB filter	0.0	0.0	5.0	5.0	0.0	0.0	0.0	0.0
Ethylhexyl salicylate/UVB filter	0.0	0.0	0.0	0.0	10.0	10.0	0.0	0.0

Legend: EHMC—ethylhexyl methoxycinnamate; OCT—octocrylene; EHS—ethylhexyl salicylate; RA—rosmarinic acid; q.s.—enough quantity to.

**Table 2:** In vitro photoprotective efficacy of sunscreen systems: in vitro SPF and critical wavelength (nm) (n = 3).

Sunscreen Systems	In Vitro SPF	Critical Wavelength (nm)
EHMC	13.7 ± 0.6 ^A^	339.7 ± 1.2 ^A^
EHMC-RA	17.0 ± 2.7 ^B^	340.0 ± 0.0 ^A^
OCT	5.7 ± 0.6 ^C,D^	347.7 ± 1.2 ^B^
OCT-RA	5.0 ± 0.0 ^C,D^	349.7 ± 1.2 ^B^
EHS	4.0 ± 0.0 ^D^	326.3 ± 0.6 ^C^
EHS-RA	7.0 ± 1.0 ^C^	331.3 ± 2.5 ^D^
Blank	1.0 ± 0.0 ^E^	N/A
Blank-RA	1.0 ± 0.0 ^E^	N/A

Legend: EHMC—ethylhexyl methoxycinnamate; OCT—octocrylene; EHS—ethylhexyl salicylate; RA—rosmarinic acid; SPF—sun protection factor; N/A—not applicable. Letters indicate differences among samples. Means not sharing same letter within same column are statistically different.

**Table 3:** Ex vivo anti-lipoperoxidative efficacy of sunscreen systems established by HPLC-TBARS-EVSC protocol (n = 8). Percentage difference between area under curve (AUC) of non-irradiated stratum corneum and AUC of irradiated and treated SC with different samples (repeated measures ANOVA with Tukey adjustment).

Variables	Percentage Difference (Mean ± Standard Deviation)	*p*-Value
SC—Blank	85.5 ± 63.2	0.782
SC—EHMC	58.0 ± 92.7	
SC—Blank	85.5 ± 63.2	0.081
SC—EHMC-RA	−4.3 ± 36.1	
SC—Blank	85.5 ± 63.2	0.349
SC—OCT	19.0 ± 54.1	
SC—Blank	85.5 ± 63.2	0.036
SC—OCT-RA	−0.32 ± 26.1	
SC—Blank	85.5 ± 63.2	0.363
SC—EHS	27.4 ± 62.4	
SC—Blank	85.5 ± 63.2	0.090
SC—EHS-RA	0.30 ± 31.4	

SC—stratum corneum non-treated and non-irradiated; EHMC—ethylhexyl methoxycinnamate; OCT—octocrylene; EHS—ethylhexyl salicylate; RA—rosmarinic acid.

## Data Availability

The data presented in this study are available on request from the corresponding author due to ethical reasons.

## Supplementary Materials

The following supporting information can be downloaded at: https://www.mdpi.com/article/10.3390/antiox14030274/s1, Table S1: Percentage difference between the AUC (area under the curve) of irradiated stratum corneum (SC) and the AUC of the different formulations (repeated measures ANOVA with Tukey adjustment).
