# Supplementary material for: Enhancing Photoprotection and Mitigating Ex Vivo Stratum Corneum Oxidative Stress: A Multifunctional Strategy Combining Rosmarinic Acid with UVB Filters
Source: Antioxidants (Basel). 2025 Feb 26;14(3):274. doi: 10.3390/antiox14030274 (PMC11939370; doi:10.3390/antiox14030274)
Supplement: Supplementary file 1 [file antioxidants-14-00274-s001.zip › antioxidants-3486819-supplementary.pdf]

Supplementary material

Table S1. Percentage difference between the AUC (area under the curve) of irradiated stratum corneum (SC) and the AUC of the different formulations (repeated measures ANOVA with Tukey adjustment).

Variables	Percentage difference mean (standard deviation)	*p*-value
Baseline irradiated SC – Blank	-27.7 (22.9)	0.009
Baseline irradiated SC – Blank - RA	-59.9 (18.7)	
Baseline irradiated SC – EHMC	-34.0 (21.3)	0.610
Baseline irradiated SC – EHMC - RA	-56.2 (29.3)	
Baseline irradiated SC – OCT	-45.3 (42.7)	0.944
Baseline irradiated SC – OCT - RA	-59.4 (15.2)	
Baseline irradiated SC – EHS	-48.3 (23.1)	0.847
Baseline irradiated SC – EHS - RA	-59.4 (13.6)	
